# Automatic behavior and communication due to pramipexole

**DOI:** 10.1186/s40734-016-0038-7

**Published:** 2016-07-11

**Authors:** Ruth H. Walker

**Affiliations:** Department of Neurology, James J. Peters Veterans Affairs Medical Center, 130 W. Kingsbridge Road, Bronx, NY 10468 USA; Department of Neurology, Mount Sinai School of Medicine, New York City, NY USA

**Keywords:** Parkinson’s disease, Automatic behavior, Dopamine agonist

## Abstract

A 45–year-old woman reported automatic behaviors and communication whilst she was being treated with pramipexole. These episodes vanished after the medication was tapered and she was started on levodopa/carbidopa. I hypothesize that the episodes were related to disordered awareness due to sleep disruption related to this medication.

## Background

Automatic behaviors [1] and “unrelated communication interludes” (UCIs) [2] have recently been reported in small numbers of patients with Parkinson’s disease (PD) during treatment with dopamine agonists. These episodes involved inappropriate actions, such as putting non-food items in the refrigerator, or the insertions of inappropriate phrases during spoken or written communication. These episodes have been attributed to excessive daytime sleepiness or other forms of sleep disruption.

Automatic behaviors are generally recognized as being typical of narcolepsy and temporal lobe epilepsy, and the phenomenon is likely best classified as a disorder of awareness [3]. These episodes are challenging to study in the laboratory setting, but are probably associated with disruption of normal sleep cycles, for example, intrusions of rapid eye movement (REM) or non-REM (NREM) sleep into wakefulness [3]. Various other disorders of consciousness (sleep attacks [4]) and behaviors (impulse control disorders, punding [5]) are well-known to be associated with the use of dopamine agonists. Automatic behaviors are likely under-recognized in PD and under-reported, especially if they consist of normal daily activities.

## Case presentation

A 45-year old, right-handed woman, who worked as a social worker, was being treated for PD which affected predominantly her right limbs. She did not report a REM sleep behavior disorder. She was unable to tolerate ropinirole due to nausea, and was switched to pramipexole. On pramipexole 1.5 mg p.o. tid she reported an episode for which she was completely amnestic. This event occurred at about 11.30pm. She had been painting a small piece of furniture, and intended to save the remaining paint to give another coat on a later day. She reported that she entered the kitchen intending to wash out her paint brush, but subsequently, without awareness, emptied and washed the 1 quart (0.95L) paint can (which was still 2/3 full) in the kitchen sink. When she again became aware of her surroundings, she found that the paint can was completely empty and had been cleaned, as had the sink, although there was paint splashed on the floor. She did not fall to the ground and her husband was asleep elsewhere in the house and did not hear anything. The patient estimated the duration of this episode, gauged by the time it would have taken to clean the paint can and the sink, was approximately 10 minutes.

The patient also reported several instances where she suddenly changed the topic and style of communication. This happened both during spoken conversation and when writing emails. For example, she completed emails to a friend and to a colleague in an inappropriately formal manner. She could not recall why she did this, but on one occasion was able to note that she felt “weird”. She describes another episode, which occurred while she was composing an email to the author thus:

“As I was gathering information for this email on 12/18 I experienced another Automatic Behavior. It was at 4:25am, so actually the morning of 12/19. I hadn’t gone to bed yet, as I had social work CEUs [Continuing Education Units] to complete. I was typing the above sentence about my husband saying to me “Hey XXXX” but instead of me completing the sentence correctly I switched subjects and the sentence read like this, “Hey, XXXX, you were talking about work and now you are talking about *Incarceration at Marin County.* It was very strange having it happen to me while I was writing to you about it, but I was definitely extremely tired at the time.” [Italics indicate inserted text].

She reported poor nighttime sleep since increasing the pramipexole, getting only 4 h sleep/night, and feeling very sleepy during the day. She did not report other cognitive issues, and was able to perform reasonably at her management-level job. There were no visual hallucinations or misperceptions. There was no evidence of any impulse control disorder. Formal neuropsychological testing was unremarkable apart from mild relative impairments in simple directed auditory attention, phonemic fluency, non-dominant left hand extremity strength, and acquisition and retention of new visual information.

Pramipexole was decreased to 0.75mg t.i.d. for one week with the addition of carbidopa-levodopa 25/100 ½ tab t.i.d. and then stopped, and carbidopa/levodopa increased to 1 tab 25/100 t.i.d. On stopping pramipexole she developed marked anxiety and panic attacks, consistent with dopamine agonist withdrawal syndrome (DAWS) [6]. She was restarted on pramipexole 0.75mg t.i.d. and tapered the medication over 5 weeks without recurrence of anxiety. She reported a marked improvement in daytime sleepiness and in nocturnal sleep disruption, and her automatic behaviors ceased.

## Conclusions

Automatic behaviors [1], and UCIs [2] appear to be newly-recognized phenomena in PD, and are likely related to disordered sleep. It is probable that these types of episodes are under-reported due to embarrassment, or unrecognized if they are congruent with appropriate behaviors or communications. UCIs were reported in 8 men [2], and automatic behaviors in five women [1], however, one of the patients in the second series also had UCIs. The current report indicates that both phenomena are aspects of the same process.

Interestingly, 2 of the initial cases were relatively young (43-52 years), comparable with the age of the current patient. These patients reported activities such as putting non-food items in the refrigerator, and putting other items in inappropriate places, and performing house-work during the night. One of these patients also reported a UCI. Fragmentation of nighttime sleep was reported in 3/5 cases, in addition to other neuropsychiatric phenomena such as visual illusions and hallucinations. Cognitive assessment showed only mild deficits.

The gender differences in the presentations of automatic behaviors in the small series previously reported [1, 2] are intriguing, however I hypothesize that this is attributable to the predominant activities of the individuals reported. The male subjects with UCIs were mainly professionally employed in roles which involved communications [2], thus their inappropriate comments were apparent in business meetings and other professional situations involving communications. The patients with motor automatic behaviors were all female [1]. Their professions are not stated, and it appears that they were working in their homes performing housekeeping tasks. Most of their automatic behaviors involved placing items in inappropriate locations, thus were readily identifiable. I hypothesize that both types of automatic activities, whether motor or communication-based, are manifestations of the same phenomenon, with ascertainment depending upon the setting.

Sleep studies of PD patients with and without sleep attacks have documented both sleep-onset REM, and sleep onset stage 2 NREM sleep [7, 8]. Some authors have proposed that sleep attacks in PD are narcolepsy-like, and it is of note that narcolepsy is the predominant disorder in which automatic behaviors are reported [3]. It was not possible to perform sleep studies in the current patient as it was necessary to taper the pramipexole as rapidly as tolerated.

Dopamine agonists have been consistently responsible for behavioral issues and disorders of sleep in PD, to a much greater extent that levo-dopa, suggesting that targeting of specific dopamine receptors, most likely the dopamine D2 receptor, might be causative. It is of note that the present patient developed pharmacologic side effects on dopamine agonists, and additionally developed DAWS on medication withdrawal. This suggests that there may be common risk factors for these complications of medications. Switching to levodopa appears to resolve the issues.

## Ethics approval

Ethics approval for this case report was waived by the Insitutional Review Board of the James J. Peter VA Medical Center.

## Consent

Written informed consent was obtained from the patient for publication of this case report and any accompanying images. A copy of the written consent is available for review by the Editor-in-Chief of this journal.

## Availability of data and supporting materials

There are no additional data or supporting materials.

## Abbreviations

DAWS, dopamine agonist withdrawal syndrome; NREM, non-rapid eye movement; PD, Parkinson’s disease; REM,rapid eye movement; UCI, unintended communication interlude
